# Biomarker-Specific Survival and Medication Cost for Patients With Non–Small Cell Lung Cancer

**DOI:** 10.1001/jamanetworkopen.2025.14519

**Published:** 2025-06-10

**Authors:** Juanyi Tan, Szu-Chun Yang, Michaela A. Dinan, Anne C. Chiang, Cary P. Gross, Shi-Yi Wang

**Affiliations:** 1 Department of Chronic Disease Epidemiology, Yale School of Public Health, New Haven, Connecticut; 2 Cancer Outcomes, Public Policy, and Effectiveness Research (COPPER) Center, Yale School of Medicine, New Haven, Connecticut; 3 Department of Internal Medicine, National Cheng Kung University Hospital, College of Medicine, National Cheng Kung University, Taiwan; 4 Department of Internal Medicine, Yale School of Medicine, New Haven, Connecticut

## Abstract

**Importance:**

Targeted therapies and immunotherapies prolong survival but are associated with high costs for patients with advanced non–small cell lung cancer (aNSCLC). To date, little is known about survival and medication cost by biomarker status in the US.

**Objective:**

To estimate survival and medication cost by aNSCLC biomarker status.

**Design, Setting, and Participants:**

This retrospective cohort study using Flatiron Health data identified patients diagnosed with aNSCLC from January 1, 2016, through December 31, 2022. Each patient had received at least 1 biomarker test and 1 documented line of therapy; follow-up was through September 31, 2023. Patients were categorized based on the presence of driver alterations, including *ALK* rearrangement, *BRAF* variation, or *EGFR* variation. Patients without driver alterations were divided into 3 groups based on their programmed cell death 1 ligand 1 (PD-L1) expression (<1%, 1%-49%, or ≥50%).

**Main Outcomes and Measures:**

The primary outcome was medication costs, which were a function of survival probability and monthly medication costs. The secondary outcome was medication costs per survivor, defined as the mean aggregate medication costs within each patient cohort for each 1- or 2-year survivor.

**Results:**

The study cohort consisted of 26 635 patients with aNSCLC (mean [SD] age at diagnosis, 68.9 [10.0] years; 13 750 [52%] male; 2610 [10%] African American, 687 [3%] Asian, 18 352 [69%] White, and 4986 [19%] other race, including any race other than African American, Asian, or White). The median overall survival was 39.9 (95% CI, 33.9-48.5) months for patients with *ALK* rearrangement, 27.0 (95% CI, 24.8-28.8) months for *EGFR* variation, 18.7 (95% CI, 16.0-20.6) months for *BRAF* variation, 12.3 (95% CI, 12.0-12.7) months for PD-L1 less than 1%, 13.7 (95% CI, 13.1-14.3) months for PD-L1 of 1% to 49%, and 16.2 (95% CI, 15.3-17.0) months for PD-L1 of 50% or greater. The 1- and 2-year medication costs per patient for the overall cohort were $120 420 (95% CI, $115 540-$126 470) and $182 560 (95% CI, $172 900-$196 040), respectively. Patients with *EGFR* variation or PD-L1 of 50% or greater incurred relatively higher 1-year medication cost ($131 700 [95% CI, $125 340-$138 280] and $123 590 [95% CI, $115 970-$130 840], respectively) compared with patients with PD-L1 less than 1% ($110 350 [95% CI, $101 680-$120 040]). Patients with *ALK* rearrangement or *EGFR* variation incurred the highest 2-year medication cost ($242 130 [95% CI, $206 220-$267 330] and $241 940 [95% CI, $230 840-$254 730], respectively), whereas patients with PD-L1 less than 1% and PD-L1 of 1% to 49% had the lowest 2-year medication cost ($156 340 [95% CI, $142 450-$172 800] and $163 410 [95% CI, $152 410-$174 180], respectively). The medication costs per 1-year survivor were $152 370 (95% CI, $133 550-$178 080) for patients with *ALK* rearrangement, $175 720 (95% CI, $167 330-$185 390) for *EGFR* variation, $211 100 (95% CI, $195 030-$229 400) for PD-L1 less than 1%, $210 260 (95% CI, $193 190-$226 580) for PD-L1 of 1% to 49%, and $211 630 (95% CI, $198 670-$224 210) for PD-L1 of 50% or greater, whereas the costs per 2-year survivor were $363 480 (95% CI, $314 710-$401 320) for patients with *ALK* rearrangement, $468 400 (95% CI, $441 340-$497 860) for patients with PD-L1 of 50% or greater, $460 790 (95% CI, $427 340-$494 080) for patients with PD-L1 of 1% to 49%, and $500 220 (95% CI, $456 900-$556 730) for patients with PD-L1 less than 1%.

**Conclusions and Relevance:**

In this cohort study, patients with aNSCLC with driver alterations experienced better survival and incurred lower medication costs per survivor than those without driver variation, indicating the need to develop more affordable and effective medications for patients without driver alterations.

## Key Points

**Question:**

What are the survival and medication cost for advanced non–small cell lung cancer by biomarker status?

**Findings:**

In this cohort study using data from 26 635 patients, the median overall survival was 39.9 months for *ALK* rearrangement, 27.0 months for *EGFR* variation, and 12 to 16 months for 3 programmed cell death 1 ligand 1 (PD-L1) groups. Medication cost per 1-year or 2-year survivor for patients without driver alterations was consistently higher than those with driver alterations.

**Meaning:**

These findings suggest that developing more affordable and effective medications for patients without driver alterations is needed.

## Teaser

This cohort study assesses the median overall survival and medication cost for patients with advanced non–small cell lung cancer by biomarker status.

## Introduction

Lung cancer is the leading cause of cancer deaths in the US, with 236 740 new cases of lung cancer and 130 180 deaths from lung cancer in 2022.^1^ Non–small cell lung cancer (NSCLC) is the main lung cancer type, accounting for 87% of these cases.^2^ Even though low-dose computed tomography screening is effective in detecting lung cancer at an early stage for high-risk populations,^3^ most NSCLC is still diagnosed at an advanced stage.^4^

In recent decades, advancements in targeted therapies^5^ and immunotherapy^6,7^ have changed the treatment landscape of advanced NSCLC (aNSCLC). The US Food and Drug Administration approved crizotinib as first-line treatment for patients with *ALK* (OMIM 105590)–rearranged aNSCLC in 2011 and alectinib in 2017. Likewise, first-line erlotinib and afatinib for aNSCLC with *EGFR* (OMIM 131550) variation received US Food and Drug Administration approval in 2013. A third-generation *EGFR* tyrosine kinase inhibitor, osimertinib, has been adopted as the front-line therapy since 2018.^8^ Immune checkpoint inhibitors have also become a standard modality of second-line treatment for most patients with aNSCLC since 2015 and emerged as the first-line therapy for patients with programmed cell death 1 ligand 1 (PD-L1) of 50% or greater in 2016.^9^

The overall economic burden of lung cancer in the US is substantial, ranking fourth among all cancers, with an estimated overall, national, out-of-pocket cost of $1.35 billion in 2019.^10^ Given the recent treatment developments, biomarker status influences whether patients can receive targeted therapeutic drugs or immunotherapy, thus playing a critical role in treatment selection. Several studies have examined health care expenditures for patients with aNSCLC in the US^11,12,13,14^; however, most of them did not stratify their patients by biomarker status. One study estimated the cost for patients with aNSCLC with *EGFR* variation,^11^ and another examined the cost for patients with *ALK*-rearranged aNSCLC.^14^ The sample size of both studies was small, and neither of these studies compared costs across different biomarker groups. We were aware that the costs differed by biomarker subtypes among patients with metastatic breast cancer.^15,16^ However, to our best knowledge, no study has yet assessed the medication cost associated with aNSCLC treatment by different biomarker groups. Analyzing community-based data, the current study aimed to assess the median overall survival (mOS) and the medication cost for patients with aNSCLC by biomarker status. For a specific biomarker group, the 1-year medication cost represented the mean per-person cost for the full group of patients after 1 year, including patients who died within 1 year. We also estimated the medication cost per 1-year survivor, using the 1-year medication cost divided by the survival probability at 1 year. This measure quantified the cost needed to spend to keep 1 patient alive at 1 year. We also applied a similar approach to estimate the 2-year medication cost and medication cost per 2-year survivor across biomarker groups.

## Methods

### Data Source

This retrospective cohort study used the nationwide Flatiron Health electronic health record–derived deidentified database. The study followed the Strengthening the Reporting of Observational Studies in Epidemiology (STROBE) reporting guideline.^17^ The Yale Human Investigation Committee determined that this study did not directly involve human participants; therefore, institutional review board approval and informed consent were not required.

### Cohort Selection

**Figure 1.**
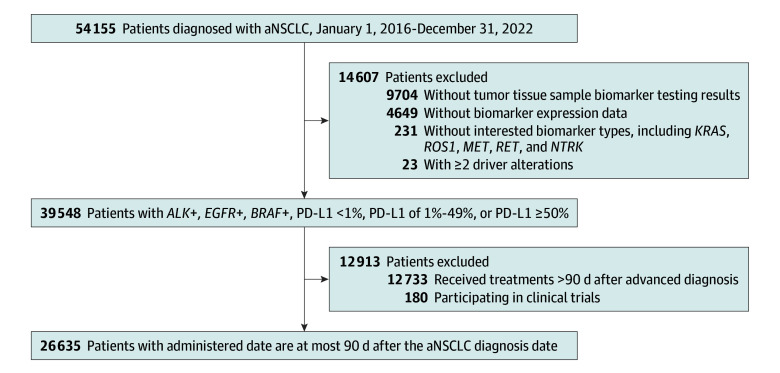
Study Cohort Selection Process *ALF*+ indicates *ALF* rearrangement; aNSCLC, advanced non–small cell lung carcinoma; *BRAF*+, BRAF variation; *EGFR*+, EGFR variation; PD-L1, programmed cell death 1 ligand 1.

Our cohort was limited to patients who were diagnosed with aNSCLC between January 1, 2016, and December 31, 2022. Patients were followed up until the last visit, death, or September 31, 2023, whichever occurred first. Each patient had received at least 1 documented line of therapy. Patients who participated in clinical trials were excluded because of absence of treatment information. To ensure the completeness of data, patients were further excluded if the first medication was administered after 90 days of aNSCLC diagnosis. We excluded patients who did not receive at least 1 biomarker test. We identified patients with driver alterations and only included 3 groups: *ALK* rearrangement, *EGFR* variation, and *BRAF* (OMIM 164757) variation. Patients who had 2 or more driver alterations were excluded. For patients without a known driver alteration, we categorized them into 3 PD-L1 groups (<1%, 1%-49%, and ≥50%). After treatment decision-making processes, we prioritized *ALK*, *EGFR*, and *BRAF* over PD-L1 information; that is, patients with known status of PD-L1 and 1 of these 3 driver alterations were categorized into the gene rearrangement or variation group. The process of the study population selection is presented in Figure 1.

### Survival and Cost Estimates

The Kaplan-Meier method was applied to estimate the mOS. The index date was the date of aNSCLC diagnosis. The event date was the death date; for those for whom a death date had not been identified, the date of last confirmed activity was used as the censored date. All drugs related to cancer treatment or to alleviating the adverse effects were identified to estimate the medication cost. In total, 815 unique drugs administered were identified through the cohort. The costs of 648 drugs were derived from the Medicare Part B and Part D dashboard.^18^ Retail costs were obtained for the remaining drugs, all of which were supportive medications and most of which had low unit prices (<$50) with limited effect on the final cost estimation.^19^

Based on a previously established method,^20,21^ we estimated the survival-adjusted monthly medication cost by multiplying the mean monthly medication cost among survivors (for each month 1 through 24) with the corresponding monthly survival probability from month 1 to month 24 after diagnosis. We then generated 2 cost estimates for each biomarker stratum. First, we estimated the 1- and 2-year medication costs for each group, summing up the survival-adjusted monthly medication cost for each month in the 12 and 24 months after aNSCLC diagnosis. We then estimated the medication costs per survivor, using the 1- and 2-year medication costs divided by the corresponding time survival probability. These measures represented the total medication costs that would be spent for each patient who survives to the milestone of 1 year and 2 years. For example, if the mean 1-year medication cost was $100 000 per person but only 50% of patients in this biomarker group survived for 1 year, then the medication cost per 1-year survivor would be $200 000. All the costs were made equivalent to 2023 US dollars using the Consumer Price Index inflation calculator^22^ and rounded at the level of $10.

### Statistical Analysis

We applied 1000 bootstrapping samples to obtain the 95% CIs of 1- and 2-year medication costs. More specifically, the 2.5th and 97.5th percentiles of the estimates were the lower and upper bounds of the 95% CIs. To estimate the additional costs these patients incurred on targeted therapies or immunotherapy, we propensity score matched 1 patient with driver alterations or positive PD-L1 with 1 patient with PD-L1 less than 1% to estimate the differences in medication cost. We selected the PD-L1 less than 1% group as the reference group because these patients were less likely to receive targeted therapy or immunotherapy. Patient characteristics accounted for by propensity score matching included age at diagnosis, year of diagnosis, sex, race and ethnicity (directly identified from the database, either self-reported or recorded by staff), geographic region, practice type, payer category, Eastern Cooperative Oncology Group (ECOG) performance status, tumor histology, and smoking status. The balances of patient characteristics between the patients with *ALK* rearrangement, *EGFR* variation, *BRAF* variation, PD-L1 of 1% to 49%, and PD-L1 of 50% or greater and the PD-L1 less than 1% propensity score–matched reference group were measured using standardized differences, which were expressed as percentages. An absolute value of less than 10 suggests that 2 groups are well balanced. Analyses were performed using SAS software, version 9.4 (SAS Institute Inc), RStudio, version 2022.07.1 + 576 (Posit PBC), and iSQoL2 package (R Foundation for Statistical Computing).

## Results

### Patient Characteristics

A total of 26 635 patients were included in our final cohort (Table 1; eTable 1 in Supplement 1). The mean (SD) age at diagnosis was 68.9 (10.0) years. Patients with *ALK*-rearranged aNSCLC were younger (mean [SD], 62.8 [13.0] years) than the other biomarker groups (all aged >67 years). A total of 13 750 patients (52%) were male and 12 885 (48%) were female. A total of 687 patients (3%) were Asian, 2610 (10%) were African American, 18 352 (69%) were White, and 4986 (19%) were other races (any race other than African American, Asian, or White). A total of 20 173 (76%) received care in community practices, 11 676 (44%) had Medicare coverage, and 11 474 (39%) had commercial health plans. A total of 9460 patients (36%) had an ECOG performance status of 0 to 1 when they were diagnosed with aNSCLC. The most common histologic subtype was nonsquamous NSCLC, accounting for 72% of the cohort (19 077 patients). Overall, there were 420 patients with *ALK* rearrangement, 840 patients with *BRAF* variation, 2249 with *EGFR* variation, 10 398 with PD-L1 less than 1%, 6422 with PD-L1 of 1% to 49%, and 6306 with PD-L1 of 50% or greater.

**Table 1.** Selected Characteristics of Patients With Advanced NSCLC, Overall and by Biomarker Status^a^

Characteristic	Overall (N = 26 635)	*ALK* (n = 420)	*BRAF* (n = 840)	*EGFR* (n = 2249)	PD-L1		
					<1% (n = 10 398)	1%-49% (n = 6422)	≥50% (n = 6306)
Age at diagnosis, mean (SD), y	68.9 (10.0)	62.8 (13.0)	68.9 (9.0)	67.9 (11.0)	69.1 (10.0)	69.2 (10.0)	69.2 (10.0)
Sex							
Female	12 885 (48)	224 (53)	424 (50)	1495 (66)	4705 (45)	2989 (47)	3048 (48)
Male	13 750 (52)	196 (47)	416 (50)	754 (34)	5693 (55)	3433 (54)	3258 (52)
Race							
African American	2610 (10)	27 (6)	77 (9)	191 (9)	1098 (11)	620 (10)	597 (10)
Asian	687 (3)	27 (6)	16 (2)	234 (10)	197 (2)	103 (2)	110 (2)
White	18352 (69)	285 (68)	597 (71)	1366 (61)	7251 (70)	4455 (70)	4398 (70)
Other race^b^	4986 (19)	81 (19)	150 (18)	458 (20)	1852 (18)	1244 (20)	1201 (19)
Ethnicity							
Hispanic	506 (2)	11 (3)	16 (2)	78 (4)	181 (2)	111 (2)	109 (2)
Non-Hispanic	26 129 (98)	409 (97)	824 (98)	2171 (96)	217 (98)	6311 (98)	6197 (98)
Community practice	20173 (76)	275 (66)	609 (73)	1561 (69)	7572 (73)	5051 (79)	5105 (81)
ECOG performance status							
0	2003 (8)	39 (9)	58 (7)	215 (10)	731 (7)	470 (7)	490 (8)
1	7457 (28)	147 (35)	243 (29)	684 (30)	2772 (27)	1818 (28)	1793 (28)
2	7530 (28)	78 (19)	243 (29)	568 (25)	2918 (28)	1919 (30)	1804 (29)
≥3	5867 (22)	70 (17)	172 (21)	412 (18)	2390 (23)	1410 (22)	1413 (22)
Unknown	3778 (14)	86 (21)	124 (15)	370 (17)	1587 (15)	805 (13)	806 (13)
Histologic subtype							
Nonsquamous NSCLC	19077 (72)	388 (92)	734 (87)	2142 (95)	7210 (69)	4133 (64)	4470 (71)
NSCLC NOS	1088 (4)	11 (3)	40 (5)	32 (1)	427 (4)	243 (4)	335 (5)
Squamous cell carcinoma	6470 (24)	21 (5)	66 (8)	75 (3)	2761 (27)	2046 (32)	1501 (24)

Abbreviations: ECOG, Eastern Cooperative Oncology Group; NOS, not otherwise specified; NSCLC, non–small cell lung cancer; PD-L1, programmed cell death 1 ligand 1.

^a^ Data are presented as number (percentage) of patients unless otherwise indicated.

^b^ Other race indicates any race other than African American, Asian, or White.

### Survival 

The mOS was 14.8 (95% CI, 14.4-15.2) months for the entire cohort, although it differed substantially across biomarker subgroups (Table 2). Patients with *ALK* rearrangement had the highest mOS (39.9 [95% CI, 33.9-48.5] months), followed by patients with *EGFR* variation (27.0 [95% CI, 24.8-28.8] months). Patients with PD-L1 less than 1% and PD-L1 of 1% to 49% had shorter mOS (12.3 [95% CI, 12.0-12.7] and 13.7 [95% CI, 13.1-14.3] months, respectively). The mOS for patients with PD-L1 of 50% or greater was 16.2 (95% CI, 15.3-17.0) months.

**Table 2.** Median Overall Survival and 1-Year and 2-Year Differences in Medication Cost by Biomarker Status^a^

Biomarker	Median overall survival (95% CI), mo	Cost difference (95% CI), $	
		1 Year	2 Years
*ALK* rearrangement	39.9 (33.9 to 48.5)	5390 (−15 780 to 29 720)	69 220 (12 320 to 103 140)
*BRAF* variation	18.7 (16.0 to 20.6)	12 410 (−5700 to 34 480)	22 930 (−6850 to 51 860)
*EGFR* variation	27.0 (24.8 to 28.8)	17 310 (3640 to 30 100)	74 130 (46 800 to 97 200)
PD-L1 1%-49%	13.7 (13.1 to 14.3)	6170 (−6100 to 19 070)	8950 (−9530 to 26 020)
PD-L1 ≥50%	16.2 (15.3 to 17.0)	13 600 (620 to 24 650)	32 380 (13 800 to 49 300)
PD-L1 <1% (reference)	12.3 (12.0 to 12.7)	NA	NA
All	14.8 (14.4 to 15.2)	NA	NA

Abbreviations: NA, not applicable; PD-L1, programmed cell death 1 ligand 1.

^a^ Results were based on propensity score–matched analyses comparing each group with patients with the reference group (PD-L1 <1%).

### Medication Cost

**Figure 2.**
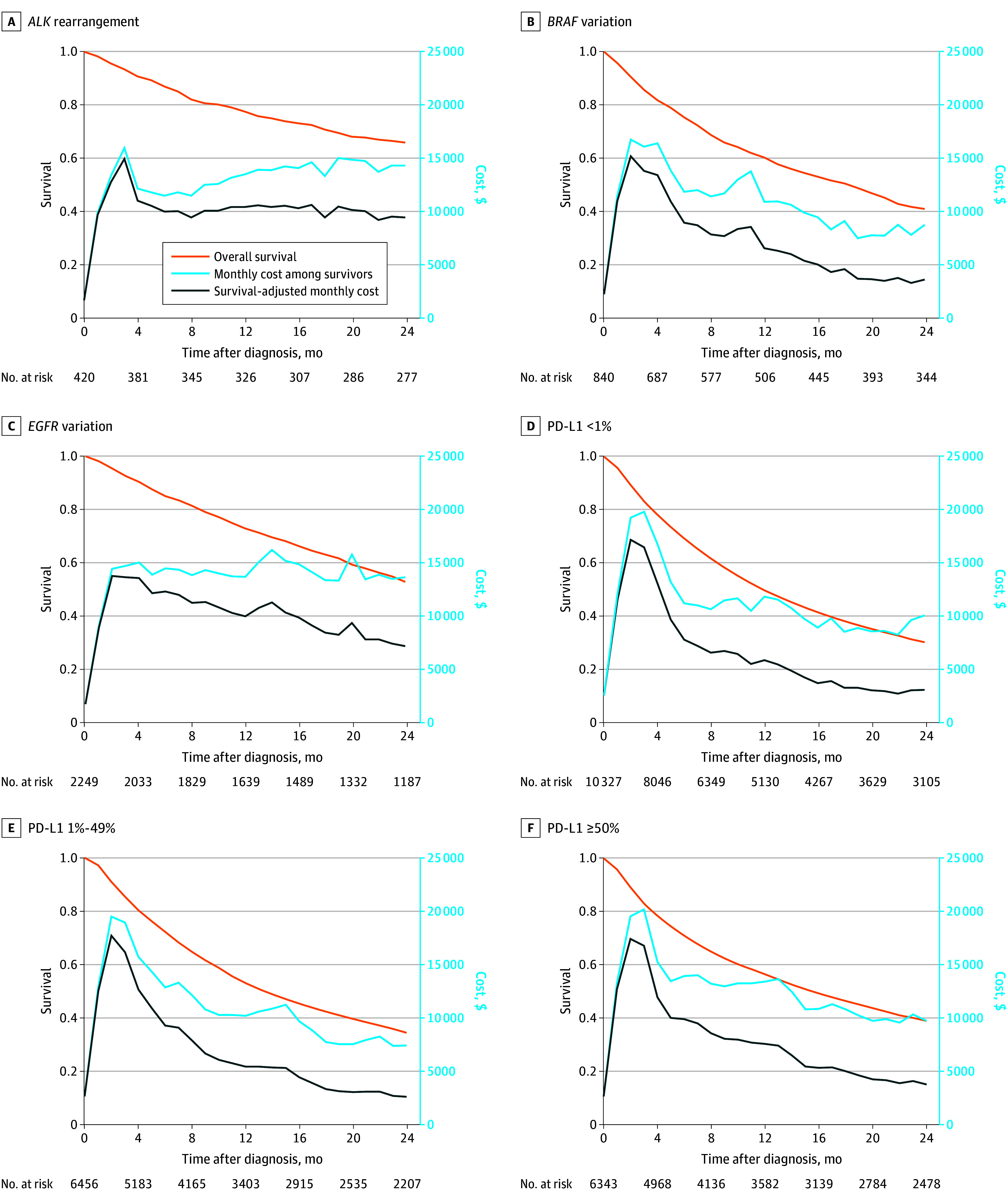
Survival, Monthly Cost Among Survivors, and Survival-Adjusted Monthly Cost by Biomarker Status PD-L1 indicates programmed cell death 1 ligand 1.

The survival and pattern of monthly medication cost among survivors in the 24 months differed by biomarker group (Figure 2). For patients with *ALK* rearrangement and *EGFR* variation, monthly medication cost among survivors was similar across month 2 through month 24. In contrast, monthly medication cost among survivors of the *BRAF* variation and the 3 PD-L1 groups reached the peak at month 2 through month 4 and then decreased over time. Additionally, the pattern of survival-adjusted monthly medication cost for patients with *ALK* rearrangement and *EGFR* variation differed from the other 4 groups (Figure 2). Survival-adjusted monthly medication cost in month 13 through month 24 in the *ALK* rearrangement and *EGFR* variation were relatively consistent, whereas those in the latter groups decreased substantially.

**Figure 3.**
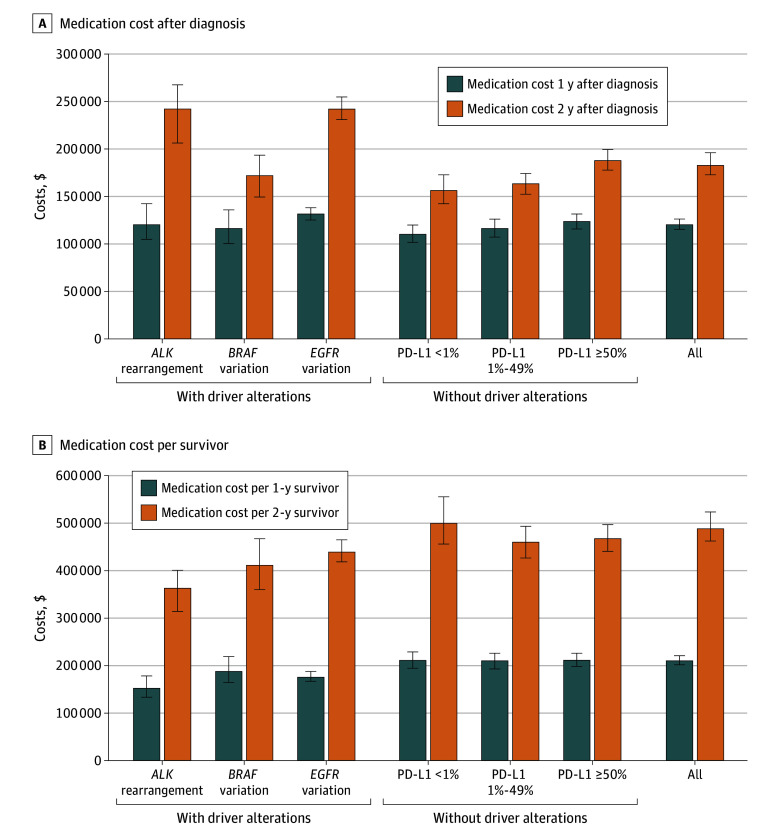
Mean 1- and 2-Year Medication Cost and Mean Medication Cost per 1-Year and 2-Year Survivor, Overall and by Biomarker Status Error bars indicate 95% CIs; PD-L1, programmed cell death 1 ligand 1.

The 1- and 2-year medication costs per patient for the overall cohort were $120 420 (95% CI, $115 540-$126 470) and $182 560 (95% CI, $172 900-$196 040), respectively (Figure 3A). Patients with *EGFR* variation or PD-L1 of 50% or greater incurred relatively higher 1-year medication cost ($131 700 [95% CI, $125 340-$138 280] and $123 590 [95% CI, $115 970-$130 840], respectively) compared with patients with PD-L1 less than 1% ($110 350 [95% CI, $101 680-$120 040]). Furthermore, patients with *ALK* rearrangement or *EGFR* variation incurred the highest 2-year medication cost ($242 130 [95% CI, $206 220-$267 330] and $241 940 [95% CI, $230 840-$254 730], respectively), whereas patients with PD-L1 less than 1% and PD-L1 of 1% to 49% had the lowest 2-year medication cost ($156 340 [95% CI, $142 450-$172 800] and $163 410 [95% CI, $152 410-$174 180], respectively).

### Medication Cost per 1-Year and 2-Year Survivors

The medication cost per 1-year and 2-year survivors also varied significantly based on biomarker status (Figure 3B). For the 1-year treatment period, patients without driver alterations required higher medication cost per survivor than patients with driver alterations. Medication costs per 1-year survivor were similar for patients with PD-L1 of 50% or greater ($211 630 [95% CI, $198 670-$224 210]), PD-L1 of 1% to 49% ($210 260 [95% CI, $193 190-$226 580), and PD-L1 less than 1% ($211 100 [95% CI, $195 030-$229 400]). Meanwhile, patients with *ALK* rearrangement or *EGFR* variation had the lowest medication cost per survivor at $152 370 (95% CI, $133 550-$178 080) and $175 720 (95% CI, $167 330-$185 390), respectively.

Similar trends were observed for the medication cost per 2-year survivor. The 3 groups of patients without driver variation had similarly high cost: PD-L1 of 50% or greater required $468 400 (95% CI, $441 340-$497 860), patients with PD-L1 of 1% to 49% required $460 790 (95% CI, $427 340-$494 080), and patients with PD-L1 less than 1% required $500 220 (95% CI, $456 900-$556 730). The medication cost per survivor was the lowest in patients with *ALK* rearrangement ($363 480 [95% CI, $314 710-$401 320]).

### Differences in Medication Cost

After propensity score matching, patient characteristics were well balanced (eTable 2 in Supplement 1). Compared with patients with PD-L1 less than 1%, matched patients with *EGFR* variation and PD-L1 of 50% or greater had significantly higher 1-year medication cost, with an estimated cost difference of $17 310 (95% CI, $3640-$30 100) and $13 600 (95% CI, $620-$24 650), respectively (Table 2). For 2-year medication cost, patients with *ALK* rearrangement, *EGFR* variation, and PD-L1 of 50% or greater had significantly higher cost than their matched PD-L1 less than 1% reference group. For example, the cost difference at 2 years after diagnosis between patients with *EGFR* variation and those with PD-L1 less than 1% was $74 130 (95% CI, $46 800-$97 200).

## Discussion

Our study is the first study, to our knowledge, to investigate the medication cost for patients with aNSCLC by biomarker status. In contrast to prior studies which only estimated the cost for patients who survived,^11,12,13,14^ we estimated the medication cost incurred by each biomarker group in 1 year and 2 years after diagnosis, accounting for monthly survival probabilities. The results showed that medication cost varied across biomarker groups. Furthermore, propensity score–matched analyses demonstrated that patients with *EGFR* variation incurred the highest 1-year medication cost, and patients with *ALK* rearrangement and *EGFR* variation incurred the highest 2-year medication cost. Our findings reflect practices in the US between 2016 and 2023, capturing the current advancement in aNSCLC treatments.

Our findings have several important clinical implications. First, our measure, medication cost per survivor, is complementary to directly estimating cost. Medication costs reflected the cost to treat patients with aNSCLC within a prespecified timeframe, whereas our measure was able to answer the question, “On average, how much do we need to treat patients with aNSCLC to keep one patient surviving for 2 years?” For instance, on average, we needed to spend $363 480 to keep 1 patient with *ALK*-rearranged aNSCLC alive for 2 years, yet we needed $500 220 for patients with PD-L1 less than 1%. Such a low medication cost per 2-year survivor in the former group was mainly associated with the fact that targeted therapies are available and effective, leading to better survival. By integrating cost estimates with survival probabilities, our measure could identify patient populations with high medication cost per survivor, for whom there is a need to develop affordable and effective treatments, as well as apply to patients with other cancer or diseases.

Second, our propensity score–matched analyses showed that patients with PD-L1 of 50% or greater had significantly higher 1- and 2-year survival-adjusted medication costs than patients with PD-L1 less than 1%. Patients with PD-L1 of 50% or greater had better overall survival and treatment response; thus, more patients would receive expensive immunotherapy, leading to higher medication cost than patients with PD-L1 less than 1%. Similarly, better overall survival and maintenance use of new-generation targeted therapies among patients with *EGFR* variation could explain why these patients had higher 1- and 2-year medication costs than patients with PD-L1 less than 1%.

Third, our complementary approaches can provide actionable information from the perspective of individuals as well as population. For instance, the 1-year costs translate to the mean medication costs for all patients in a given subgroup during the first year after diagnosis, recognizing that some may not live a full year. Conversely, the per-survivor costs provide a useful way to understand the aggregate investment in cancer therapeutics for each 1-year survivor. By integrating costs with effectiveness, medication cost per 1-year survivor can quantify the amount needed to spend to keep 1 patient alive at 1 year. Collectively, these costs are more comprehensive for estimating costs for a population than simply identifying the patients who survived for 1 year and tallying their costs. This latter approach, estimating costs from survivors only, is likely to overestimate costs. The magnitude of overestimation would become more prominent when the survival was poor or the timeframe increased. For instance, estimates derived from the survivors would overestimate 1-year and 2-year costs for patients with ALK rearrangement by 20% and 26% and for patients with PD-L1 less than 1% by 56% and 116%, respectively (eTable 3 in Supplement 1).

The survival outcomes of our study cohort were worse than those from clinical trials, such as the KN-189 trial (mOS of 22 months for *EGFR-* and *ALK*-negative patients),^23^ FLAURA trial (mOS of 38.6 months for patients with *EGFR* variation),^24^ and ALEX trial (mOS of >57.4 months for patients with *ALK* rearrangement treated with alectinib).^25^ Some reasons could help explain this discrepancy. First, we included patients with poor ECOG status. Second, many of our patients did not receive an optimal treatment regimen. Third, quality of care might be better in clinical trials. Indeed, a recent study of patients with *ALK* rearrangement using the Flatiron data demonstrated a similar result as our study.^26^

### Limitations

We acknowledge limitations in our study. First, the current cost estimation is limited to cancer-related, outpatient medication cost. Cost of inpatient stays, surgery, radiation, imaging, and laboratory testing were not included in our analysis. Second, we acknowledged that medication prices are plan dependent and differ by region, and manufacturer discounts and rebates affect medication costs.^27^ We used Medicare drug reimbursement rates for cost estimation, which could reflect societal cost because this approach has been applied in economic evaluation in the US.^16,28^ Because not all drugs administered to patients in the cohort were covered by Medicare, we used retail prices to complement the missing information. This issue should not have a substantial effect on our findings given that the costs of these drugs were small. Third, although lines of therapies have been an important aspect in measuring costs in oncology, our cost estimates included all medication costs within 1 year and 2 years, regardless of the lines of therapies. Examining biomarker-specific costs by individual first line of therapy is beyond the scope of this study. Future research is needed. Fourth, although 1- and 2-year medication costs accounted for survival probability, we did not adjust for patient characteristics. The estimates, however, are consistent with the findings derived from cost differences based on propensity score–matched analyses. Fifth, because the sample sizes of patients with other driver alterations, such as *MET*, *NTRK*, *RET*, and *ROS1*, were too small, we did not explore the cost associated with these patients.

## Conclusions

In this retrospective cohort study, overall survival and medication cost associated with aNSCLC treatment varied across biomarker groups. Compared with those without driver alterations, patients with driver alterations experienced better survival. One-year medication cost was the highest for patients with *EGFR* variation, and 2-year medication cost was the highest for patients with *ALK* rearrangement. Medication cost per 1-year or 2-year survivor for patients without driver alterations was consistently higher than those with driver alterations, indicating the need to develop cheaper or more effective medications for these patients. This study offers up-to-date insights into the medication cost for patients with aNSCLC by biomarker status, which could facilitate future economic evaluation of precision medicine in aNSCLC.

## Notes

Supplement 1. **eTable 1.** Characteristics of Patients With Advanced Non–Small Cell Lung Cancer, Overall and by Biomarker Status **eTable 2.** Standardized Mean Differences as Compared With the Matched PD-L1 <1% Reference Group **eTable 3.** Difference Between 1-Year/2-Year Medication Costs Derived From the Method Accounting for Survival Probability and Medication Costs Derived From 1-Year/2-Year Survivors

Supplement 2.
Data Sharing Statement
