# Supplementary material for: Biomarker-Specific Survival and Medication Cost for Patients With Non–Small Cell Lung Cancer
Source: JAMA Netw Open. 2025 Jun 10;8(6):e2514519. doi: 10.1001/jamanetworkopen.2025.14519 (PMC12152704; doi:10.1001/jamanetworkopen.2025.14519)
Supplement: Supplement 1. — eTable 1. Characteristics of Patients With Advanced Non–Small Cell Lung Cancer, Overall and by Biomarker Status eTable 2. Standardized Mean Differences as Compared With the Matched PD-L1 <1% Reference Group eTable 3. Difference Between 1-Year/2-Year Medication Costs Derived From the Method Accounting for Survival Probability and Medication Costs Derived From 1-Year/2-Year Survivors [file jamanetwopen-e2514519-s001.pdf]

Supplemental Online Content

Tan J, Yang S-C, Dinan MA, Chiang AC, Gross CP, Wang S-Y. Biomarker-specific survival and medication cost for patients with non–small cell lung cancer *JAMA Netw Open*. 2025;8(6):e2514519. doi:10.1001/jamanetworkopen.2025.14519

eTable 1. Characteristics of Patients With Advanced Non–Small Cell Lung Cancer, Overall and by Biomarker Status

eTable 2. Standardized Mean Differences as Compared With the Matched PD-L1 <1% Reference Group

eTable 3. Difference Between 1-Year/2-Year Medication Costs Derived From the Method Accounting for Survival Probability and Medication Costs Derived From 1-Year/2-Year Survivors

This supplemental material has been provided by the authors to give readers additional information about their work.

© 2025 Tan J et al. *JAMA Network Open.*

eTable 1. Characteristics of patients with advanced non–small cell lung cancer, overall and by biomarker status

	Overall *n* = 26,635	*ALK* *n* = 420	*BRAF* *n* = 840	*EGFR* *n* = 2249	PD-L1 <1% *n* = 10,398	PD-L1 1%-49% *n* = 6422	PD-L1 ≥50% *n* = 6306
Age at diagnosis, mean (SD)	68.9 (10)	62.8 (13)	68.9 (9)	67.9 (11)	69.1 (10)	69.2 (10)	69.2 (10)
Year of diagnosis							
2016-2017	6375 (24)	148 (35)	175 (21)	634 (28)	2607 (25)	1289 (20)	1522 (24)
2018-2020	12,608 (47)	179 (43)	390 (46)	1034 (46)	4746 (46)	3125 (49)	3134 (50)
2021-2022	7652 (29)	93 (22)	275 (33)	581 (26)	3045 (29)	2008 (31)	1650 (26)
Sex, n (%)							
Female	12,885 (48)	224 (53)	424 (50)	1495 (66)	4705 (45)	2989 (47)	3048 (48)
Male	13,750 (52)	196 (47)	416 (50)	754 (34)	5693 (55)	3433 (54)	3258 (52)
Race, n (%)							
Asian	687 (3)	27 (6)	16 (2)	234 (10)	197 (2)	103 (2)	110 (2)
African American	2610 (10)	27 (6)	77 (9)	191 (9)	1098 (11)	620 (10)	597 (10)
Other Race*	4986 (19)	81 (19)	150 (18)	458 (20)	1852 (18)	1244 (20)	1201 (19)
White	18,352 (69)	285 (68)	597 (71)	1366 (61)	7251 (70)	4455 (70)	4398 (70)
Hispanic, n (%)	506 (2)	11 (3)	16 (2)	78 (4)	181 (2)	111 (2)	109 (2)
Region, n (%)							
Midwest	2953 (11)	38 (9)	95 (11)	162 (7)	1156 (11)	736 (12)	766 (12)
Northeast	3545 (13)	52 (12)	120 (14)	297 (13)	1301 (13)	870 (14)	905 (14)
Southeast	8969 (34)	108 (26)	253 (30)	573 (26)	3395 (33)	2314 (36)	2326 (37)
Southwest	2450 (9)	33 (8)	81 (10)	211 (9)	993 (10)	614 (10)	518 (8)
West	1643 (6)	33 (8)	46 (6)	225 (10)	513 (5)	383 (6)	443 (7)
Unknown	7075 (27)	156 (37)	245 (29)	781 (35)	3040 (29)	1505 (23)	1348 (21)
Practice type, n (%)							
Community	20,173 (76)	275 (66)	609 (73)	1561 (69)	7572 (73)	5051 (79)	5105 (81)
Payer category, n (%)							
Commercial Health Plan	10474 (39)	201 (48)	313 (37)	917 (41)	4053 (39)	2555 (40)	2435 (39)
Medicaid	2946 (11)	42 (10)	82 (10)	215 (10)	1173 (11)	735 (11)	699 (11)
Medicare	11,676 (44)	144 (34)	397 (47)	997 (44)	4584 (44)	2774 (43)	2780 (44)
Others	1539 (6)	33 (8)	48 (6)	120 (5)	588 (6)	358 (6)	392 (6)
ECOG performance status, n (%)							
0	2003 (8)	39 (9)	58 (7)	215 (10)	731 (7)	470 (7)	490 (8)
1	7457 (28)	147 (35)	243 (29)	684 (30)	2772 (27)	1818 (28)	1793 (28)
2	7530 (28)	78 (19)	243 (29)	568 (25)	2918 (28)	1919 (30)	1804 (29)

© 2025 Tan J et al. *JAMA Network Open.*

3+	5867 (22)	70 (17)	172 (21)	412 (18)	2390 (23)	1410 (22)	1413 (22)
Unknown	3778 (14)	86 (21)	124 (15)	370 (17)	1587 (15)	805 (13)	806 (13)
Histology, n (%)							
Non-squamous cell carcinoma	19077 (72)	388 (92)	734 (87)	2142 (95)	7210 (69)	4133 (64)	4470 (71)
NSCLC histology NOS	1088 (4)	11 (3)	40 (5)	32 (1)	427 (4)	243 (4)	335 (5)
Squamous cell carcinoma	6470 (24)	21 (5)	66 (8)	75 (3)	2761 (27)	2046 (32)	1501 (24)
Smoking status, n (%)							
History of smoking	23,408 (88)	210 (50)	743 (89)	1204 (54)	9483 (91)	5912 (92)	5856 (93)
No history of smoking	3200 (12)	209 (50)	96 (11)	1044 (46)	902 (9)	503 (8)	446 (7)
Unknown/Not documented	27 (0)	1 (0)	1 (0)	1 (0)	13 (0)	7 (0)	4 (0)

ECOG: Eastern Cooperative Oncology Group; NSCLC NOS: non-small cell lung cancer not otherwise specified; SD: standard deviation.

* Other race: Race other than White, African American, and Asian was grouped as other race.

© 2025 Tan J et al. *JAMA Network Open.*

eTable 2. Standardized mean differences as compared with the matched PD-L1 <1% reference group

	ALK-rearrangement *n* = 420	*BRAF*-variation *n* = 840	*EGFR*-variation *n* = 2055	PD-L1 1-49% *n* = 6456	PD-L1 ≥50% *n* = 6343
Age at diagnosis					
<55	2.20	2.11	-0.78	-0.19	0.94
55-60	-5.00	0.74	3.99	0.37	-0.32
60-65	-1.37	-1.33	5.68	0.30	0.22
65-70	0.00	-0.30	1.46	0.49	0.80
70-75	4.96	0.92	1.68	0.72	-0.85
≥75	-1.16	-1.04	-8.60	-1.35	-0.40
Diagnosis year					
2016-2017	-5.93	-0.88	-0.33	0.19	1.67
2018-2020	4.35	3.59	3.42	-0.06	0.35
2021-2022	1.73	-3.03	-3.51	-0.10	-1.99
Sex					
Female	1.43	0.48	4.64	0.93	1.61
Male	-1.43	-0.48	-4.64	-0.93	-1.61
Race					
African American	-2.84	-0.82	4.96	1.21	0.92
Asian or other race*	-2.17	8.67	7.42	0.58	1.87
White	3.55	-6.95	-9.76	-1.28	-2.24
Region					
Midwest	1.68	-2.59	-3.23	0.68	0.87
Northeast	4.45	0.68	-0.14	0.86	1.08
Southeast	-3.77	-2.07	-1.54	-0.61	-1.04
Southwest	0.89	2.46	1.99	-0.21	1.44
West	3.64	0.53	1.20	-0.59	0.50
Unknown	-2.95	1.58	1.44	-0.04	-1.65
Histology					
Non-squamous NSCLC or NSCLC NOS	2.14	0.00	-1.86	-1.84	-0.19
Squamous cell carcinoma	-2.14	0.00	1.86	1.84	0.19
ECOG performance status					

© 2025 Tan J et al. *JAMA Network Open.*

0-1	7.23	-0.99	6.43	-1.65	-1.21
>=2	-2.48	-0.48	-8.01	1.12	-1.14
Unknown	-5.78	2.03	2.39	0.70	3.53
Smoking status					
History of smoking	0.00	-3.04	-1.78	-2.72	-2.04
No history of smoking	0.00	3.04	1.78	2.49	2.04
Unknown/Not documented	0.00	0.00	0.00	2.49	0.00
Payer category					
Commercial health plan	-9.06	-6.35	-0.89	-0.47	-0.29
Medicaid	15.98	4.13	6.79	2.69	0.30
Medicare	-0.50	0.00	-4.69	-1.93	-0.32
Others	1.79	8.95	3.96	1.57	0.86
Practice type					
Academic	-4.48	0.80	-0.11	0.08	-1.65
Community	4.48	-0.80	0.11	-0.08	1.65

ECOG: Eastern Cooperative Oncology Group; NSCLC NOS: Non-small cell lung cancer not otherwise specified.

* Other race: Race other than White, African American, and Asian was grouped as other race.

© 2025 Tan J et al. *JAMA Network Open.*

eTable 3. Difference between 1-year/2-year medication costs derived from the method accounting for survival probability and medication costs derived from 1-year/2-year survivors

	1-year medication cost, $	Medication cost, derived from survivors, $	Difference
***ALK*-rearrangement**	120,540	144,950	20%
***BRAF*-variation**	116,330	188,860	62%
***EGFR*-variation**	131,700	162,400	23%
**PD-L1 <1%**	110,350	171,730	56%
**PD-L1 1-49%**	116,380	168,560	45%
**PD-L1 ≥50%**	123,590	176,100	42%
**All**	120,420	171,010	42%

	2-year medication cost, $	Medication cost, derived from survivors, $	Difference
***ALK*-rearrangement**	242,130	306,190	26%
***BRAF*-variation**	172,020	321,280	87%
***EGFR*-variation**	241,940	338,400	40%
**PD-L1 <1%**	156,340	332,310	113%
**PD-L1 1-49%**	163,410	294,010	80%
**PD-L1 ≥50%**	187,800	322,910	72%
**All**	182,560	337,420	85%

© 2025 Tan J et al. *JAMA Network Open.*
