# Supplementary material for: Biomarker-Specific Survival and Medication Cost for Patients With Non–Small Cell Lung Cancer
Source: JAMA Netw Open. 2025 Jun 10;8(6):e2514519. doi: 10.1001/jamanetworkopen.2025.14519 (PMC12152704; doi:10.1001/jamanetworkopen.2025.14519)
Supplement: Supplement 2. — Data Sharing Statement [file jamanetwopen-e2514519-s002.pdf]

Data Sharing Statement

Tan. Biomarker-Specific Survival and Medication Cost for Patients With Non–Small Cell Lung Cancer. *JAMA Netw Open*. Published June 10, 2025.
doi:10.1001/jamanetworkopen.2025.14519

Data
Data available: No

Additional Information
Explanation for why data not available: The data that support the findings of this study were originated by and are the property of Flatiron Health, Inc., which has restrictions prohibiting the authors from making the data set publicly available. Requests for data sharing by license or by permission for the specific purpose of replicating results in this manuscript can be submitted to PublicationsDataAccess@flatiron.com.
